# A Ratio Model of *L*_1_/*L*_2_ Norm for Sound Source Identification

**DOI:** 10.3390/s20185290

**Published:** 2020-09-16

**Authors:** Linsen Huang, Zhongming Xu, Zhifei Zhang, Yansong He

**Affiliations:** School of Automotive Engineering, Chongqing University, 174 Shezhengjie, Chongqing 400044, China; 20183201008@cqu.edu.cn (L.H.); z.zhang@cqu.edu.cn (Z.Z.); hys68@cqu.edu.cn (Y.H.)

**Keywords:** microphone array, near-field acoustical holography, sound source identification, sparse regularization

## Abstract

In the field of sound source identification, robust and accurate identification of the targeted source could be a challenging task. Most of the existing methods select the regularization parameters whose value could directly affect the accuracy of sound source identification during the solving processing. In this paper, we introduced the ratio model $\ell_{1}/\ell_{2}$ norm to identify the sound source(s) in the engineering field. Using the alternating direction method of multipliers solver, the proposed approach could avoid the selection of the regularization parameter and localize sound source(s) with robustness at low and medium frequencies. Compared with other three methods employing classical penalty functions, including the Tikhonov regularization method, the iterative zoom-out-thresholding algorithm and the fast iterative shrinkage-thresholding algorithm, the Monte Carlo Analysis shows that the proposed approach with $\ell_{1}/\ell_{2}$ model leads to stable sound pressure reconstruction results at low and medium frequencies. The proposed method demonstrates beneficial distance-adaptability and signal-to-noise ratio (SNR)-adaptability for sound source identification inverse problems.

## 1. Introduction

The acoustic imaging techniques have played a central role in the Noise, Vibration, and Harshness (NVH) engineering field of sound source identification, such as automotive, aircraft, and industrial applications [1,2,3]. As one of the excellent acoustic imaging tools, near-field acoustical holography (NAH) was first formalized four decades ago by the groundbreaking work of Williams [4,5]. It is an experimental method that estimates a complex sound field, based on the sound pressure measured by a microphone array close to the measured object. NAH gradually becomes an ideal technique which provides high spatial resolution for sound source localization and quantification. NAH is often cast as an acoustic reconstruction problem. However, both the resolution quality and the economic cost in NAH are limited by the size of sampling dataset.

The findings of compressive sensing (CS) techniques shed new light on the microphone array signal processing [6]. Much literature has examined the combined sparse regularization strategy based on the CS to deal with the time- and labor-intensive tasks in the community of acoustic imaging. Chardon [7] firstly introduced the CS technique into NAH to reduce the number of samplings and tuned the data fidelity constraint by using a function of the hologram norm. In the research conducted by Attendu [8], the author predicted the optimal regularization parameter with the Pareto frontier curve which is another kind of the L-curve approach. Fernandez-Grande [9] estimated a noise floor, assuming that the signal-to-noise ratio (SNR) is known. Gerstoft [10] set out to examine the regularization parameter selection via the least absolute shrinkage and selection operator (LASSO) path. Hald [11] tested a simple method about the determination of the regularization parameter which is based on a specified dynamic range in decibels. Giri [12] applied the regularized least squares (RLS) method as a baseline method to heuristically choose the regularization parameter. Hu [13] adopted the general cross validation to determine the regularization parameter in the proposed method. Shi [14] found a reasonable weighting parameter of the objective function through many experiments. Bi [15] determined the best value parameter in the data fidelity constraint by the 10-fold cross validation method. Tan [16] proposed an approach that allows the penalty to be automatically adjusted by putting the measured distance into the regularization parameter. However, the methods selecting the regularization parameter are involved in these above-mentioned reports and most of them are heuristic. To that end, an objective approach for the performance evaluation should be designed, so that it could be free from the experimental conditions. The work by Hald [11] mentioned that the selection of the regularization parameter could be avoided by solving the constrained $\ell_{1}$ minimization problem. Correspondingly, Rahimi [17] proposed a ratio of $\ell_{1}$ and $\ell_{2}$ norms model denoted as $\ell_{1}/\ell_{2}$ which is comparable to other approaches in sparse recovery. Pham [18] mapped a sparse moving-source with a smoothed $\ell_{1}/\ell_{2}$ regularization method. Wang [19] connected the relationship between $\ell_{1}/\ell_{2}$ model and $\ell_{1}-\alpha \ell_{2}$ model in the domain of sparse recovery. Enlighten by the scale-invariant $\ell_{1}/\ell_{2}$ norm ratio model, we intend to introduce this ratio model into the application of sound source identification to avoid the selection of the regularization parameter during the solving process.

In this presented paper, an approach using the ratio model of the $\ell_{1}$ and $\ell_{2}$ norm (denoted as $\ell_{1}/\ell_{2}$) for sound source identification is proposed. We intend to solve this problem with the alternating direction method of multipliers (ADMM). To benchmark the performance of the proposed method, we compare with other three reconstruction methods including the Tikhonov regularization method (denoted as Tikhonov), the iterative zoom-out-thresholding algorithm (IZOTA) [11] and the fast iterative shrinkage-thresholding algorithm (FISTA) [20] to demonstrate the advantage of the proposed method in the application of sound source identification.

The remainder of this paper is the following: Section 2 models the sound source reconstruction problem. Section 3 introduces the ratio model of $\ell_{1}/\ell_{2}$ norm. Section 4 evaluates the proposed method on performance assessments and error evaluations compared with Tikhonov, IZOTA, and FISTA in numerical simulations. Section 5 verifies the availability of the proposed method by experimental results, and Section 6 concludes and summarizes this paper.

## 2. Sound Source Reconstruction Model

The equivalent source method (ESM) enables the sound field generated by random sources to be interchanged by a number of equivalent sources in an assumed equivalent source plane [21,22]. With *N* equivalent sources in the equivalent source plane and *M* microphones in the microphone array (i.e., the holography plane), Equation (1) obtains the acoustic pressure at *m*th microphone:

(1) $$p\left(m\right)=\sum_{n=1}^{N}g\left(r_{m}|r_{n}\right)q_{n}$$

where $g\left(r_{m}|r_{n}\right)$ is the free-field Green’s function that establishes the relationship between *n*th equivalent source and *m*th microphone. $g\left(r_{m}|r_{n}\right)=e^{-jk∥{\vec{r}}_{mn}{∥}_{2}}/4\pi {∥{\vec{r}}_{mn}∥}_{2}$, k=2πf/c is the wavenumber (in rad/m), *f* represents the frequency and *c* means the acoustic speed (in m/s), $j=\sqrt{-1}$. $∥{\vec{r}}_{mn}{∥}_{2}$ represents the distances between the equivalent sources and the sensors. The Equation (2) relates the microphone array and the equivalent sources q.

(2) p=Aq

where p is the vector form of elements p(m); The vector A is an M×N transfer matrix. After calculating the equivalent sources strength q, the Equation (3) reconstructs this inverse problem to obtain the reconstructed acoustic pressure $p_{R}$

(3) $$p_{R}=Gq,$$

where G is the other transfer matrix which relates the equivalent source plane and the reconstruction plane. Often, Equation (2) could be solved by the Tikhonov regularization method which is a kind of $\ell_{2}$ norm type regularization method.

(4) $$\min_{q}{∥p-Aq∥}_{2}^{2}+\lambda {∥q∥}_{2}^{2}$$

where ${∥p-Aq∥}_{2}^{2}$ is known as measurement error, it could be interpreted the correctness of fitness for the liner system; ${∥q∥}_{2}^{2}$ is the regularizer with $\ell_{2}$ norm. λ is a positive regularization parameter to balance the relative importance between the measurement error and sparsity. However, the solution of the Tikhonov regularization method, $q_{\mathrm{tik}}={\left[A^{H}A+\lambda^{2}I\right]}^{-1}A^{H}p$, with I is the M×M identity matrix, is smooth rather not sparse. One strategy would be to recast the $\ell_{2}$ norm problem solved by the conventional regularization method into the $\ell_{1}$ norm minimization model by sparsity-promoting. As previously described [23], $\ell_{1}$ norm regularization frame could promote the sparsity, but it fails to generate a solution with enough sparsity.

(5) $$\min_{q}{∥p-Aq∥}_{2}^{2}+\lambda {∥q∥}_{1}$$

Another strategy would be to replace the $\ell_{1}$ norm minimization by introducing a sparsity-promoting $\ell_{0}$ norm penalty [24]. The sparse representation in Equation (2) could be transformed to Equation (6). Yet this would lead to a combinatory optimization problem; this acquires a sparse but underestimated solution via the corresponding sparse regularization model at a high computational cost.

(6) $$\min_{q}{∥q∥}_{0}\;\;s.t.\;{∥p-Aq∥}_{2}^{2}\leq \varepsilon$$

where ${∥q∥}_{0}$ is the number of nonzero components (usually named the $\ell_{0}$ norm though it is a pseudo norm), ε is the noise level. The sparse frame of solution could be measured by ${∥q∥}_{0}$. Because $\ell_{0}$ norm is a non-convex and non-smooth function, Equation (6) is a non-deterministic polynomial-time (NP) hard problem. To avoid the computational difficulty of solving the model (6), Candès [25] summarised the methods that could relax it to the $\ell_{1}$ norm frame.

## 3. The Introduction of Ratio Model of $L_{1}/L_{2}$ Norm

We applied the ratio model of the $\ell_{1}$ and $\ell_{2}$ norm for sound source identification:

(7) $$\min_{q\in C^{N}}\left\{\frac{{∥q∥}_{1}}{{∥q∥}_{2}}+{∥Aq-p∥}_{2}^{2}\right\}$$

Using the frame of decomposition-coordination, the alternating direction method of multipliers (denoted as ADMM) forms simply and functions powerfully; it solves small local subproblems to coordinate to solve a large global problem [26]. The combination of both the dual decomposition and augmented Lagrangian methods paves the way for ADMM to solve the constraint optimization problem. Therefore, we intended to solve the ratio model of the $\ell_{1}$ and $\ell_{2}$ norm via the ADMM solver and we introduced two auxiliary variables to apply the ADMM solver.

The Equation (7) could be rewritten into Equation (8) [17,27],

(8) $$\min_{q,y,z}\left\{\frac{{∥z∥}_{1}}{{∥y∥}_{2}}+{∥Aq-p∥}_{2}^{2}\right\}\;\;s.t.\;y=q,\;z=q$$

As discussed in [26], ADMM runs well without specific tuning. The augmented Lagrangian $L_{\rho }\left(q,y,z;v,w\right)$ is expressed in Equation (9) by reformulating Equation (8):

(9) $$\begin{array}{cc} L_{\rho }\left(q,y,z;v,w\right)= & \frac{{∥z∥}_{1}}{{∥y∥}_{2}}+{∥Aq-p∥}_{2}^{2}+\frac{\rho }{2}{∥q-y+\frac{1}{\rho }v∥}_{2}^{2}+\frac{\rho }{2}{∥q-z+\frac{1}{\rho }w∥}_{2}^{2} \end{array}$$

where y and z are two auxiliary variables, v and w are two Lagrange multiplier vectors. The Equation (10) processes in following five steps by updating q,y,z,v,w individually:

(10) $$\left\{\begin{array}{c} q^{\left(k+1\right)}:=\arg\min_{q}L_{\rho }\left(q,y^{\left(k\right)},z^{\left(k\right)};v^{\left(k\right)},w^{\left(k\right)}\right)\\ y^{\left(k+1\right)}:=\arg\min_{y}L_{\rho }\left(q^{\left(k+1\right)},y,z^{\left(k\right)};v^{\left(k\right)},w^{\left(k\right)}\right)\\ z^{\left(k+1\right)}:=\arg\min_{z}L_{\rho }\left(q^{\left(k+1\right)},y^{\left(k+1\right)},z;v^{\left(k\right)},w^{\left(k\right)}\right)\\ v^{\left(k+1\right)}:=v^{\left(k\right)}+\rho \left(q^{\left(k+1\right)}-y^{\left(k+1\right)}\right)\\ w^{\left(k+1\right)}:=w^{\left(k\right)}+\rho \left(q^{\left(k+1\right)}-z^{\left(k+1\right)}\right) \end{array}\right.$$

where ρ is a positive constant and it is suggested to be 200 in this study, and ${\left(\cdot \right)}^{\left(k+1\right)}$ implies the ${\left(k+1\right)}^{th}$ iteration. The update for q could be expressed in Equation (11),

(11) $$\begin{array}{c} q^{\left(k+1\right)}=\left(I-A^{T}{\left(AA^{T}\right)}^{-1}A\right)f^{\left(k\right)}+A^{T}{\left(AA^{T}\right)}^{-1}p \end{array}$$

where I refers to a diagonal matrix and $f^{\left(k\right)}=\frac{1}{2}\left(y^{\left(k\right)}-\frac{1}{\rho }v^{\left(k\right)}\right)+\frac{1}{2}\left(z^{\left(k\right)}-\frac{1}{\rho }w^{\left(k\right)}\right)$.

For the solution of the y-subproblem, let $c^{\left(k\right)}={∥z^{\left(k\right)}∥}_{1}$ and $d^{\left(k\right)}=q^{\left(k+1\right)}+\frac{v^{\left(k\right)}}{\rho }$, this subproblem could be reduced to Equation (12):

(12) $$y^{\left(k+1\right)}=\arg\min_{y}\frac{c^{\left(k\right)}}{{∥y∥}_{2}}+\frac{\rho }{2}{∥y-d^{\left(k\right)}∥}_{2}^{2}$$

For the auxiliary variable y, we could obtain Equation (13) by taking derivative of the objective function:

(13) $$\left(-\frac{c^{\left(k\right)}}{{∥y∥}_{2}^{3}}+\rho \right)y=\rho d^{\left(k\right)}$$

As a result, there exists a positive number $\tau^{\left(k\right)}\geq 0$ such that $y=\tau^{\left(k\right)}d^{\left(k\right)}$; Given $d^{\left(k\right)}$, we denote $\eta^{\left(k\right)}={∥d^{\left(k\right)}∥}_{2}$ and $D^{\left(k\right)}=\frac{c^{\left(k\right)}}{\rho {\left(\eta^{\left(k\right)}\right)}^{3}}$, then

(14) $$\tau^{3}-\tau^{2}-D^{\left(k\right)}=0$$

The Equation (14) acquires only one real root according to the cubic-root formula. The Equation (15) provides the closed-form solution.

(15) $$\tau^{\left(k\right)}=\frac{1}{3}+\frac{1}{3}\left(C^{\left(k\right)}+\frac{1}{C^{\left(k\right)}}\right)$$

with $C^{\left(k\right)}=\sqrt[{3}]{\frac{27D^{\left(k\right)}+2+\sqrt{{\left(27D^{\left(k\right)}+2\right)}^{2}-4}}{2}}$.

In summary, the update for the auxiliary variable y in Equation (9) could be expressed in Equation (16)

(16) $$y^{\left(k+1\right)}=\left\{\begin{array}{cc} e^{\left(k\right)} & d^{\left(k\right)}=0\\ \tau^{\left(k\right)}d^{\left(k\right)} & d^{\left(k\right)}\neq 0 \end{array}\right.$$

where $e^{\left(k\right)}$ is a random vector with the $\ell_{2}$ norm to be $\sqrt[{3}]{\frac{c^{\left(k\right)}}{\rho }}$.

Finally, the update for the auxiliary variable z in Equation (9) could be expressed in Equation (17):

(17) $$z^{\left(k+1\right)}=S\left(q^{\left(k+1\right)}+\frac{w^{\left(k\right)}}{\rho },\frac{1}{\rho {∥y^{\left(k+1\right)}∥}_{2}}\right)$$

where $S{\left(b,\mu \right)}_{i}$ is the soft-thresholding function as Equation (18) defined:

(18) $$S{\left(b,\mu \right)}_{i}=\mathrm{sign}\left(b_{i}\right)\max\left(0,\left|b_{i}\right|-\mu \right),i=1,2,\ldots n$$

We determined the regularization parameter in the Tikhonov regularization method via the L-curve method; followed the regularization parameter in FISTA based on the literature [11]; set the parameters in IZOTA as per the reference [28]. For FISTA and the proposed approach, the iteration is started with the initial guess $q^{\left(0\right)}$ with a vector of zeros; the maximum iteration is 600.

## 4. Simulations Analysis

We describe the technique in two main phases: performance assessments and error evaluations respectively.

**Figure 1:**
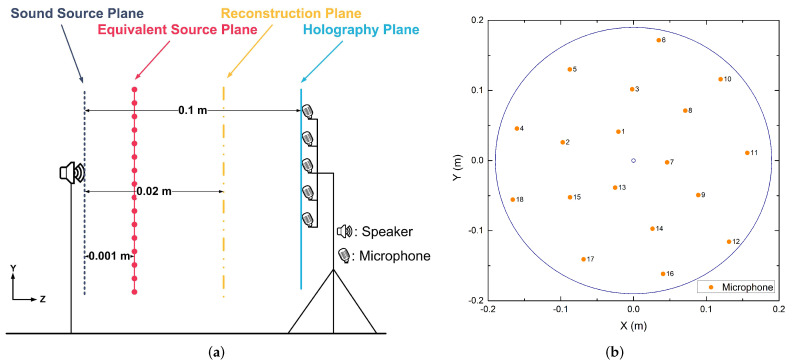
(**a**) The diagrammatic sketch of simulation set-up; (**b**) The geometry of microphone array.

In all simulations, we assumed that the sound velocity is 340 m/s; the sound source was a vibrating sphere of radius 0.01 m, which vibrated at $2.5\times 10^{-2}$ m/s. The equivalent source plane, the reconstruction plane, and the microphone array (i.e., the holography plane) were $10^{-3}$ m, $10^{-2}$ m, and 0.1 m from the sound source plane respectively (see Figure 1a); Figure 1b illustrates the geometry of 18-channel irregular microphone array. For the single sound source case, the point source was placed at (0, 0, 0) m; for the coherent sound sources case, the point sources were placed at (0.2, 0, 0) m and (−0.2, 0, 0) m respectively. The meshing grids of the equivalent source plane and the reconstruction plane were 41×41 grids whose spacing is $10^{-2}$ m. White Gaussian noise with SNR 30 dB was added to the simulations.

We acquired all the numerical results of simulation by running a MATLAB 9.2 (R2017a) implementation of four methods including Tikhonov, IZOTA, FISTA, and $\ell_{1}/\ell_{2}$ on a standard PC (8 GB of RAM on Windows 10) with CPU (Intel i5-8400, 2.8 GHz).

### 4.1. Performance Assessments

**Figure 2:**
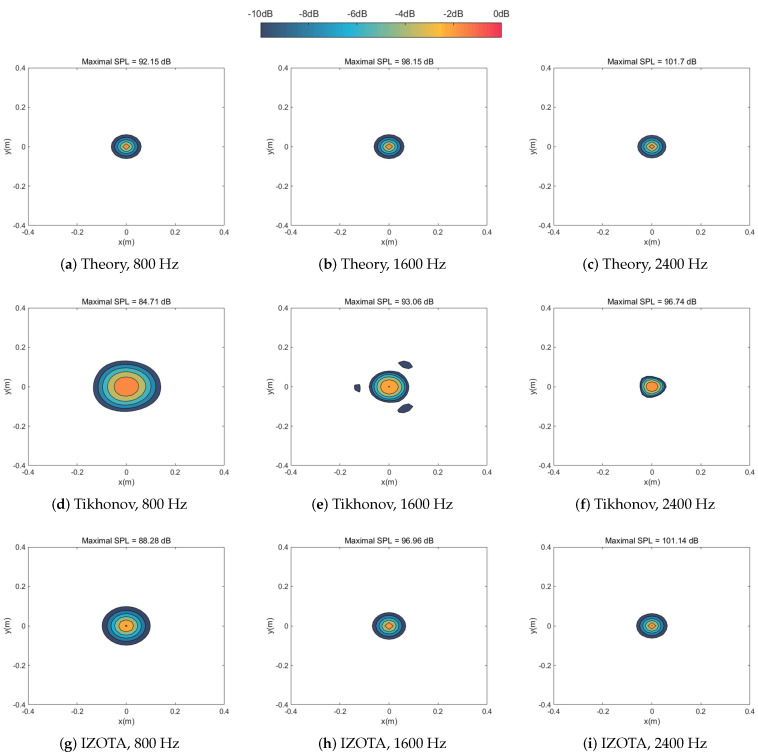

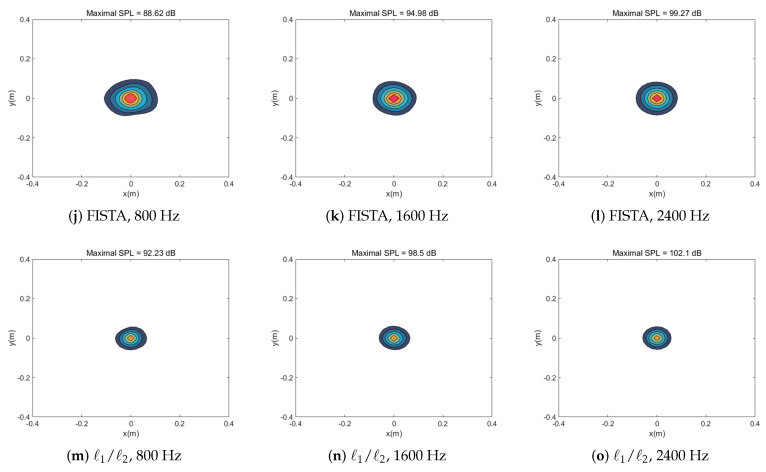
Calculated sound source maps, with signal-to-noise ratio (SNR) 30 dB, at the frequencies of 800 Hz, 1600 Hz, and 2400 Hz for single sound source respectively: the equivalent source plane at $10^{-3}$ m, the reconstruction plane at $10^{-2}$ m, and the microphone array at 0.1 m distance from the sound source plane.

**Figure 3:**
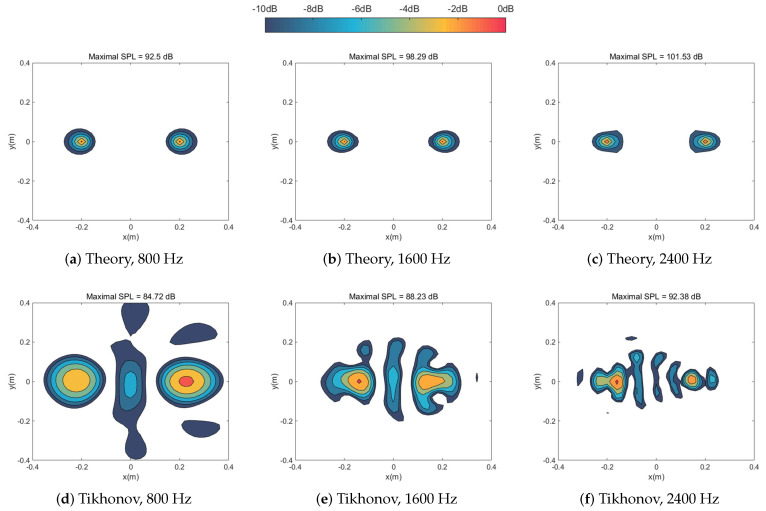

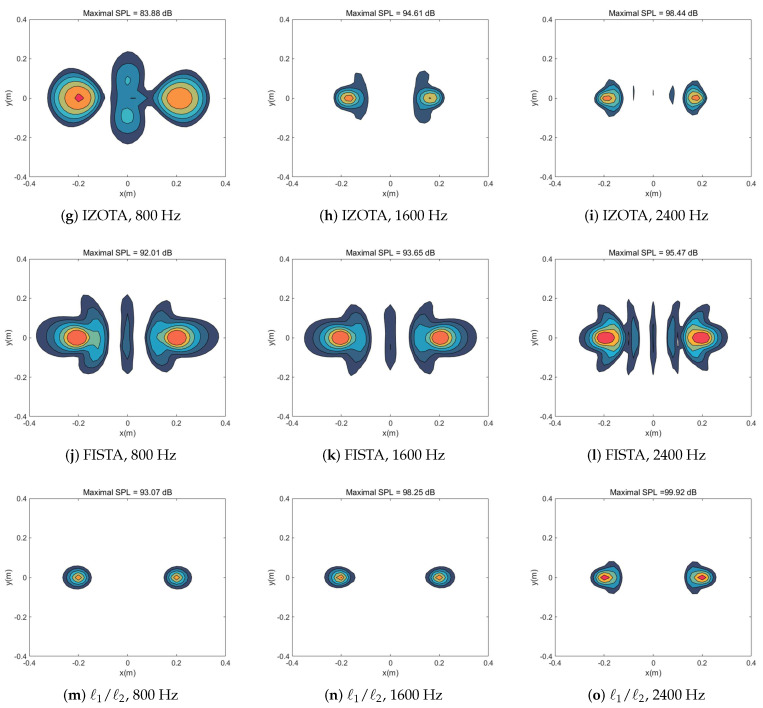
Calculated sound source maps, with SNR 30 dB, at the frequencies of 800 Hz, 1600 Hz, and 2400 Hz for coherent sound sources respectively: The equivalent source plane at $10^{-3}$ m, the reconstruction plane at $10^{-2}$ m, and the microphone array at 0.1 m distance from the sound source plane.

Figure 2 and Figure 3 simulated numerically the sound pressure reconstruction maps at the frequencies of 800 Hz, 1600 Hz, and 2400 Hz in turn for the single sound source case and the coherent sound sources case respectively. We found that the Tikhonov regularization method failed to focus on the sound source(s) since the identified acoustic pressure of the main lobe was lower than 10 dB compared with other methods and mixed the coherent sound sources into a hot-spot (Figure 3a). As a mainstream method in the field of NAH, IZOTA coincided with well the theoretical value in both of cases; it underperformed at the frequency of 800 Hz for the coherent sound sources case (Figure 3g), since this method did not provide a minimize function of the $\ell_{1}$ norm regularization problem [11]. FISTA focused on the centers but it failed to depress the side lobes effectively (Figure 3j). The proposed method detected the position of the targeted sound source(s) and reconstructed the acoustic pressure accurately compared with the theoretical value (Figure 3m).

**Figure 4:**
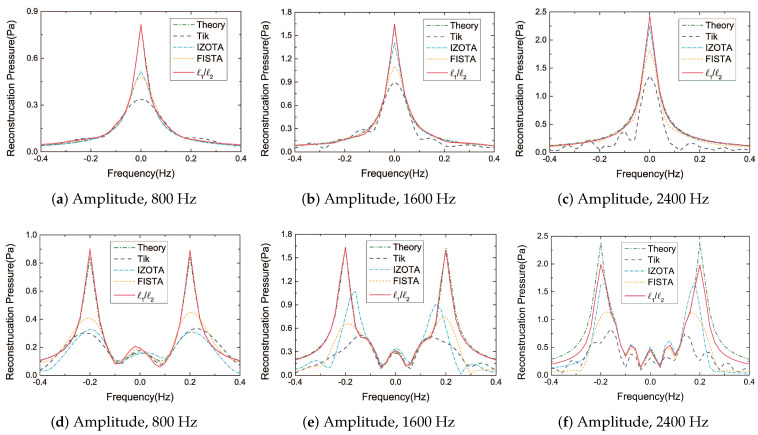
The cross-section of the reconstruction acoustic pressure plane: (**a**–**c**) are in the single sound source case, sound source at (0, 0, 0) m; (**d**–**f**) are in the coherent sound sources case, sound sources at (0.2, 0, 0) m and (−0.2, 0, 0) m: The equivalent source plane at $10^{-3}$ m, the reconstruction plane at $10^{-2}$ m, and the microphone array at 0.1 m distance from the sound source plane.

Figure 4 reveals the cross-section of the reconstruction acoustic pressure plane for both cases. The Tikhonov regularization method peaked at half of the theoretical value; IZOTA was superior to FISTA and it underperformed at low frequencies in the coherent sound sources case; the proposed method fitted the theoretical value expected in the coherent sound sources case at the frequency of 2400 Hz.

### 4.2. Error Evaluations

To quantitatively rate these four methods in percentage terms, the reconstruction error (RE) and the quantitative error (QE) were defined in Equation (19):

(19) $$\mathrm{RE}=\sqrt{\sum_{i=1}^{N}{\left(\left|p_{i}\right|-\left|{\bar{p}}_{i}\right|\right)}^{2}}/\sqrt{\sum_{i=1}^{N}{\left|{\bar{p}}_{i}\right|}^{2}};\;\mathrm{QE}=\left|\max\left(SPL_{\mathrm{theory}}\right)-\max\left(SPL_{\mathrm{reconstruction}}\right)\right|$$

where $p_{i}$ is the reconstructed value of acoustic pressure and ${\bar{p}}_{i}$ is the theoretical value of acoustic pressure; $SPL_{\mathrm{reconstruction}}$ is the reconstructed value of acoustic pressure sound pressure level (SPL) and $SPL_{\mathrm{theory}}$ is the theoretical value of sound pressure level.

**Figure 5:**
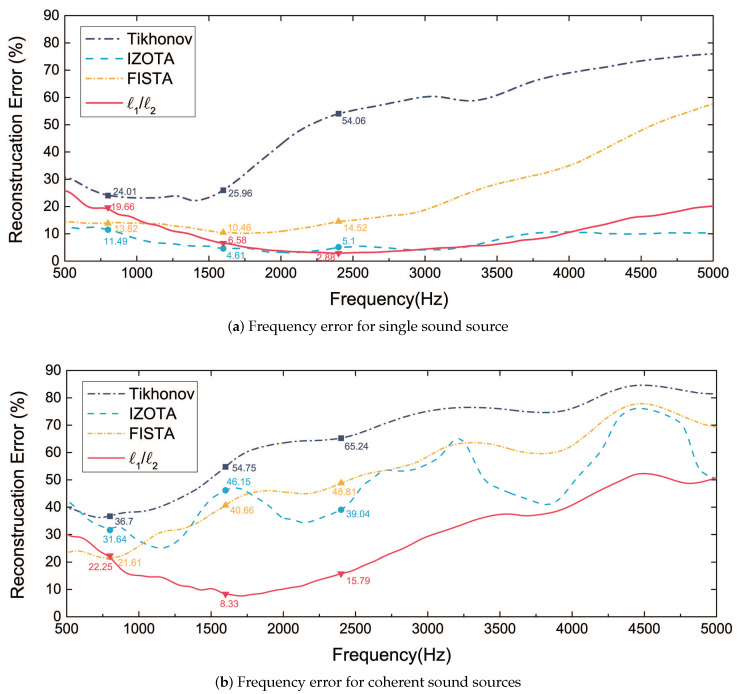
The error calculated by the Tikhonov regularization method, iterative zoom-out-thresholding algorithm (IZOTA), fast iterative shrinkage-thresholding algorithm (FISTA), and $\ell_{1}/\ell_{2}$, the equivalent source plane at $10^{-3}$ m, the reconstruction plane at $10^{-2}$ m, and the microphone array at 0.1 m distance from the sound source plane, with SNR 30 dB: (**a**) the single sound source case, sound source at (0, 0, 0) m; (**b**) the coherent sound sources case, sound sources at (0.2, 0, 0) m and (−0.2, 0, 0) m respectively.

Figure 5 depicts the error of reconstructed acoustic pressure in the frequency band from 500 Hz to 5000 Hz. The reconstruction error was performed using the average value of 30 sets of simulations. Tikhonov failed to work with the increase of frequency. The increase of RE for FISTA was the most obvious error and it was also the largest error increase among the four algorithms. It increased faster in the coherent sound sources case compared with the single sound source case. IZOTA was relatively stable (below 15%), though it fluctuated along with the number supplement of the sound source (over 30%). The proposed method performed a stable function (below 30%) at low and medium frequencies. It is clear for the reconstruction error that the proposed method could decrease below 10% along with the increase of the frequency in the monopole sound source case.

**Figure 6:**
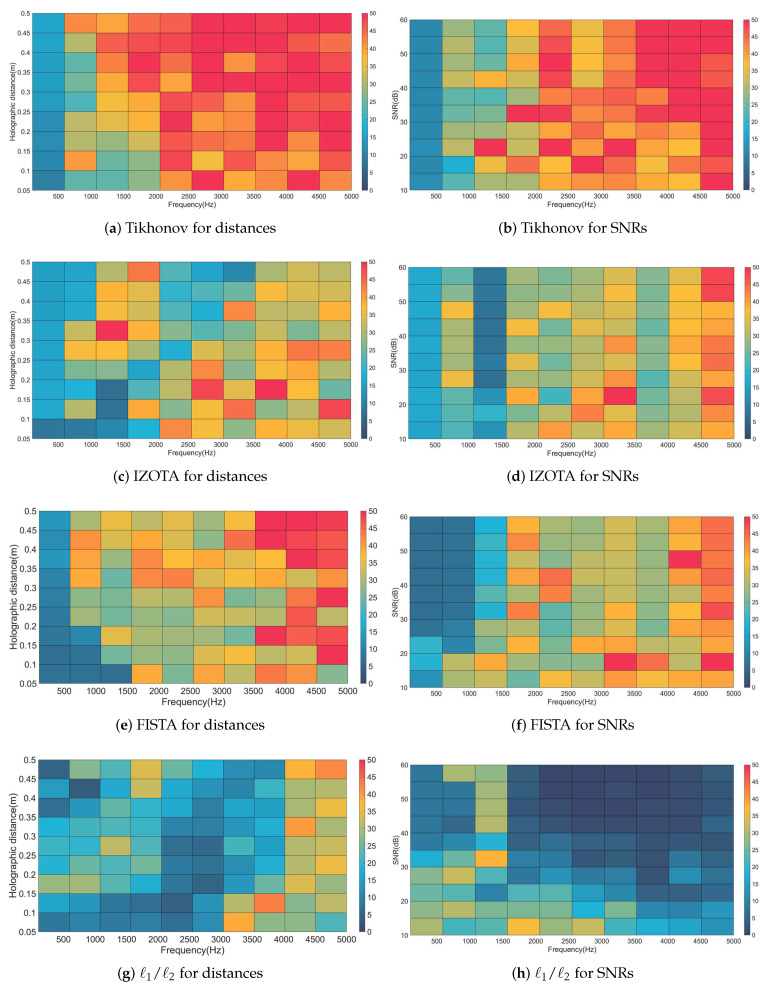
The quantitative error of methods with holographic distance 0.05∼0.5 m (**left column**), with SNR 30 dB; The quantitative error of methods with SNR 10∼60 dB (**right column**), with holographic distance 0.1 m.

Next, we adopted the quantitative error maps for the coherent sound sources case to evaluate the comprehensive performance which was influenced by different frequencies, varied measured distances, and dissimilar SNRs. Figure 6a,c,e,g presents the quantitative error (QE) of four above-mentioned algorithms in the frequency band of 500∼5000 Hz in with holographic distances of 0.05∼0.5 m and SNR 30 dB; in Figure 6b,d,f,h with SNR 10∼60 dB and holographic distance 0.1 m. The contour maps shared the same color bars whose maximum values were set to 50%.

On the distance-adaptability hand, Figure 6a shows that Tikhonov ran well in the near-field but stumbled in the high-frequency measurements. IZOTA extended the distance and the frequency for the measurement (Figure 6c). Figure 6e shows that FISTA could fail to maximize the performance when the frequency above 3500 Hz and the measured distance over 0.3 m since the quantitative error exceeded 40%. $\ell_{1}/\ell_{2}$ provided the acceptable reconstruction accuracy at low frequencies and in the near-field measurements; it is worth noting that $\ell_{1}/\ell_{2}$ could be adaptive well to the far-field measurements with the frequency increased (Figure 6g). On the SNR-adaptability hand, the QE of Tikhonov failed to be adaptive in the high-frequency band (Figure 6b). IZOTA and FISTA progressed obviously and they fitted at low and medium frequencies with SNR 10∼60 dB. $\ell_{1}/\ell_{2}$ performed well over SNR 20 dB at low and medium frequencies (Figure 6h).

On top of that, we conducted an *T*-trail Monte Carlo simulation at 350 Hz interval frequency from 450 Hz to 5000 Hz, to evaluate the reconstruction accuracy. In this Monte Carlo simulation, the position of the sound source was random while the SNR was 30 dB. According to the references [29,30,31], we defined the average reconstruction error (ARE) and the average quantitative error (AQE) in Equations (20) and (21) apiece,

(20) $$\mathrm{ARE}=\frac{1}{T}\sum_{i=1}^{T}\left(20\mathrm{lg}\left(\sum_{i=1}^{N}\left(\left|p_{i}\right|/\left|{\bar{p}}_{i}\right|\right)\right)/\left(20\mathrm{lg}\left(\sum_{i=1}^{N}\left|{\bar{p}}_{i}\right|\right)\right)\right)$$

(21) $$\mathrm{AQE}=\frac{1}{T}\sum_{i=1}^{T}\left(\frac{1}{N}\sum_{i=1}^{N}20\mathrm{lg}\left(\left|p_{i}\right|/\left|{\bar{p}}_{i}\right|\right)\right)$$

where T=1000, *N* is the number of the equivalent sources.

**Figure 7:**
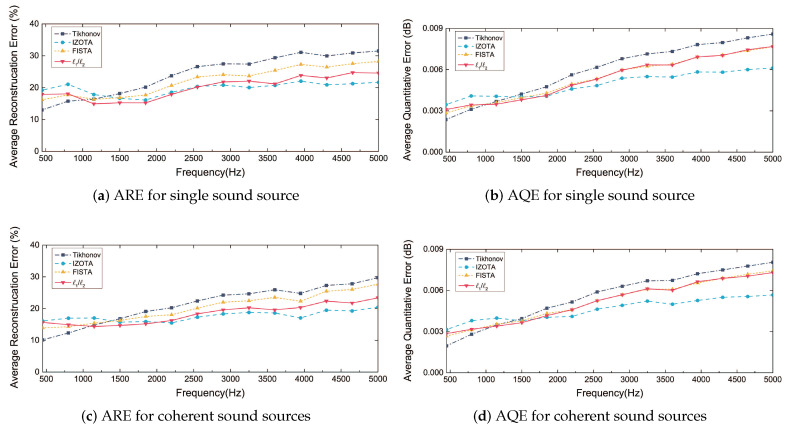
The average quantitative errors (AQE) and the average reconstruction errors (ARE) for the 1000-trial Monte Carlo analysis by the Tikhonov regularization method, IZOTA, FISTA, and $\ell_{1}/\ell_{2}$, with the position of the sound source random and SNR 30 dB.

Figure 7 shows the curves of the average reconstruction error (ARE) and the average quantitative error (AQE) calculated by the four above-mentioned methods under 1000-trial Monte Carlo simulation in both cases. The smaller value of ARE and AQE could present a better and more stable reconstruction performance. Over the 1200 Hz frequency range, IZOTA and $\ell_{1}/\ell_{2}$ stepped in steady-state while Tikhonov and FISTA were in a rising state. Below the 1200 Hz frequency range, Tikhonov was the best among the four methods.

Taken together, on the reconstruction accuracy and stability hand, IZOTA was superior to $\ell_{1}/\ell_{2}$; $\ell_{1}/\ell_{2}$ is superior to FISTA; FISTA was superior to Tikhonov. It is worth noting that, compared to IZOTA [28], $\ell_{1}/\ell_{2}$ required fewer parameters to be selected.

## 5. Experimental Applications

**Figure 8:**
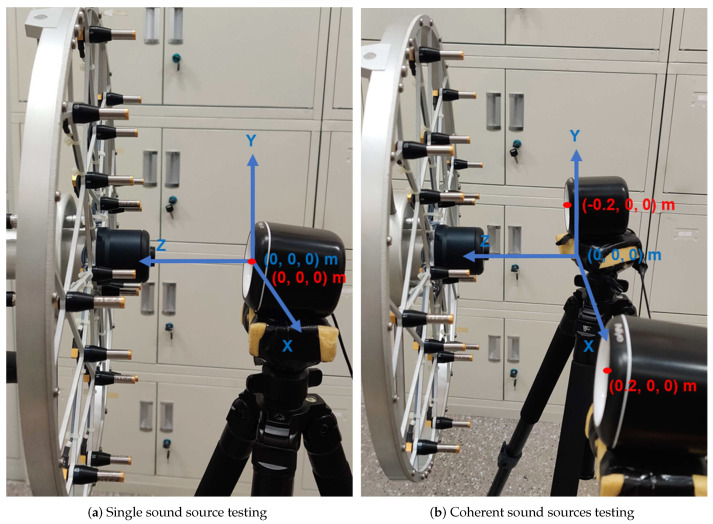
The testing set-up: (**a**) The single sound source case, sound source at (0, 0, 0) m; (**b**) The coherent sound sources case, sound sources at (0.2, 0, 0) m and (−0.2, 0, 0) m. The equivalent source plane at $10^{-3}$ m (z = $10^{-3}$ m), the reconstruction plane at $10^{-2}$ m (z = $10^{-2}$ m), and the microphone array at 0.1 m (z = $10^{-1}$ m) distance from the sound source plane.

We conducted experiments to check the ability of these methods. The same setting as simulation, we placed the speaker(s) excited by a steady-state sinusoidal acoustic wave at the targeted position (as visible in Figure 8). The speaker was at the origin of the coordinates in the single sound source case while the speakers were at (0.2, 0, 0) m and (−0.2, 0, 0) m in the coherent sound sources case. The sound source plane was the plane perpendicular to the ground on which the speaker was located. The equivalent source plane was at $10^{-3}$ m (z = $10^{-3}$ m), the reconstruction plane was at $10^{-2}$ m (z = $10^{-2}$ m), and the microphone array was at 0.1 m (z = $10^{-1}$ m) distance from the sound source plane. We acquired the data using an 18-channel Combo microphone array made by HBK company, which was a random distribution of HBK 4951 microphones in a circle of 0.38 m diameter. The sampling frequency was 32,768 Hz and the sampling time was 5 s. Collected by the HBK LAN-XI data collector, the sound pressure data were processed to the acoustic imaging via the MATLAB software. The reconstructed sound pressure map exported the sound pressure level (SPL), with 2 dB intervals and −10 dB from the peak value as the display range.

**Figure 9:**
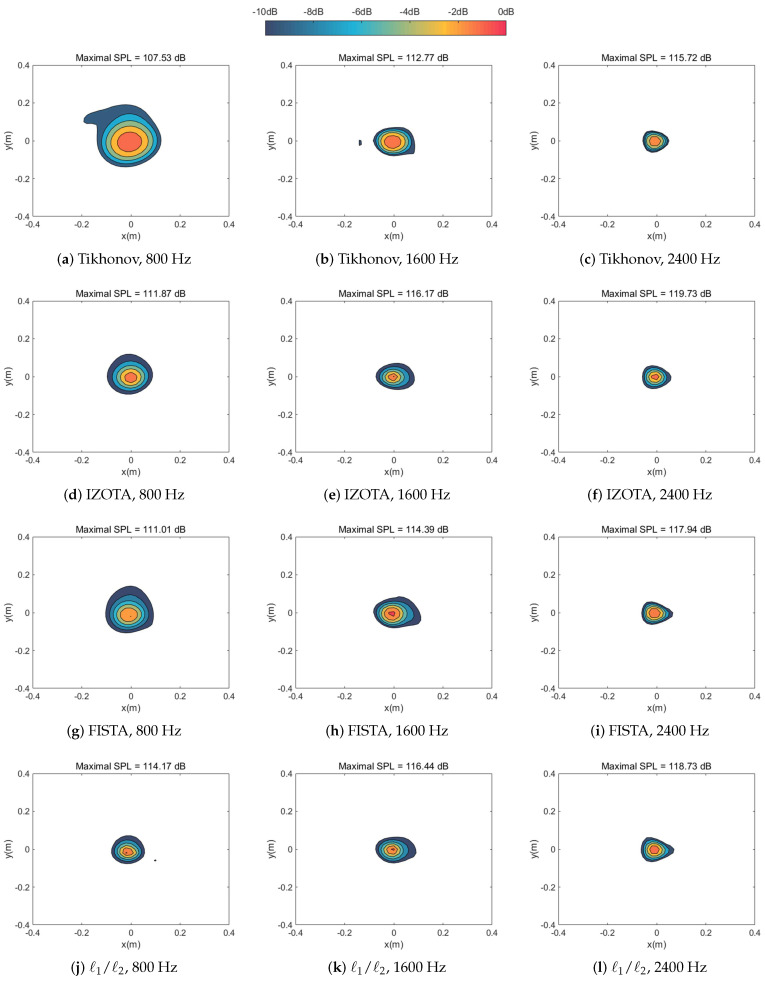
Experimental sound source maps at the frequencies of 800 Hz, 1600 Hz, and 2400 Hz respectively for single sound source case, the microphone array at 0.1 m distance from the sound source plane.

**Figure 10:**
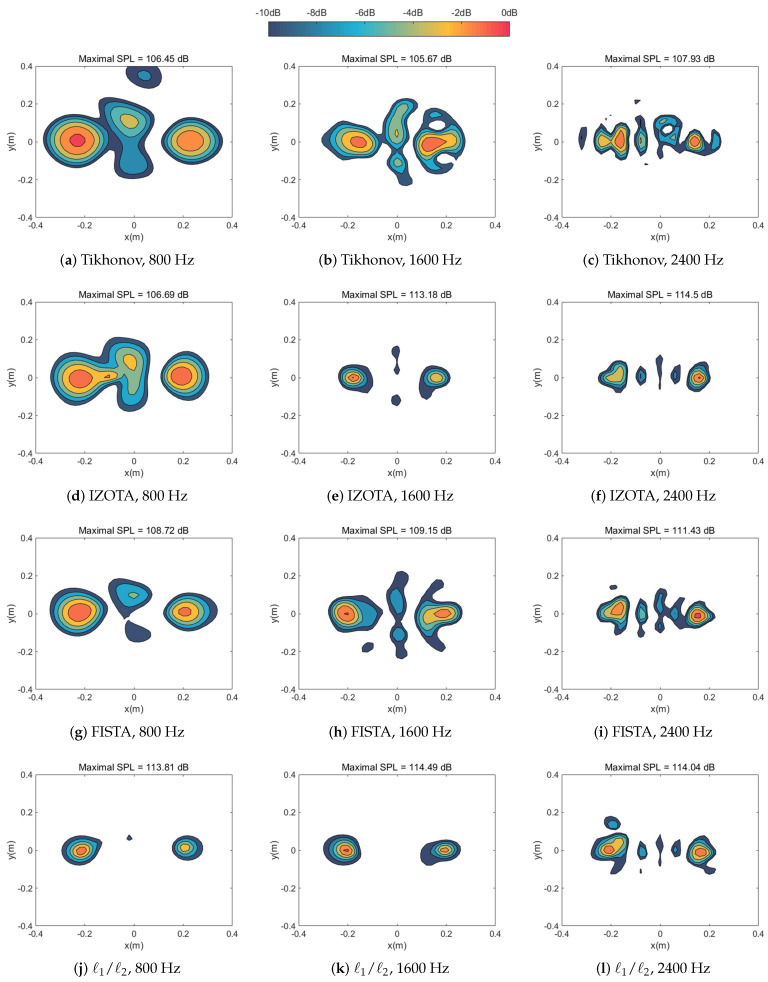
Experimental sound source maps at the frequencies of 800 Hz, 1600 Hz, and 2400 Hz respectively for coherent sound sources case, the microphone array at 0.1 m distance from the sound source plane.

Figure 9 and Figure 10 shows the experimental sound pressure maps at the frequencies of 800 Hz, 1600 Hz, and 2400 Hz in turn for the monopole sound source case and the coherent sound sources case respectively. We found that the IZOTA performed extremely well in the monopole sound source case while it failed to depress the sidelobe at low frequencies in the coherent sound sources case. FISTA corresponded to the simulation analysis results. The proposed method could detect the position of the sound source(s), separate the coherent sound sources, and reconstruct the acoustic pressure accurately.

The experimental results of this study should be considered in light of the following limitation: the experiments were conducted in a room with non-anechoic chamber conditions, so noise interference may have been present. We speculate that this was the reason for the difference between the 2400 Hz simulated and experimental results.

## 6. Conclusions

In this paper, an approach is proposed to obtain a robust and accurate estimation for sound source identification via the $\ell_{1}/\ell_{2}$ norm ratio model. Using the ADMM solver, the proposed approach could avoid the selection of the regularization parameter.

Simulation analysis results illustrate that the proposed method could recognized the location of sound source(s) and reconstruct the acoustic pressure close to the theoretical value; it localizes sound source with robustness, and expresses the distance-adaptability and SNR-adaptability at low and medium frequencies. The Monte Carlo analysis shows that the proposed approach leads to stable sound pressure reconstruction results at low and medium frequencies. Experimental applications chart the sound pressure map at the frequencies of 800 Hz, 1600 Hz, and 2400 Hz; the results indicate that $\ell_{1}/\ell_{2}$ could separate the coherent sound sources at 800 Hz, while IZOTA underperformed.

The proposed method could offer potential gains in industrial applications for sound source identification.
